# Investigation of Forced Convection Enhancement and Entropy Generation of Nanofluid Flow through a Corrugated Minichannel Filled with a Porous Media

**DOI:** 10.3390/e22091008

**Published:** 2020-09-09

**Authors:** Ehsan Aminian, Hesam Moghadasi, Hamid Saffari, Amir Mirza Gheitaghy

**Affiliations:** 1 School of Mechanical Engineering, Iran University of Science and Technology, Tehran 16846-13114, Iran; e_aminian@mecheng.iust.ac.ir (E.A.); hesam_moghadasi@mecheng.iust.ac.ir (H.M.); saffari@iust.ac.ir (H.S.); 2 Department of Microelectronics, Delft University of Technology, 2628 CD Delft, The Netherlands

**Keywords:** heat transfer, geometrical parameters, entropy generation, nanofluid, porous media

## Abstract

Corrugating channel wall is considered to be an efficient procedure for achieving improved heat transfer. Further enhancement can be obtained through the utilization of nanofluids and porous media with high thermal conductivity. This paper presents the effect of geometrical parameters for the determination of an appropriate configuration. Furthermore, the optimization of forced convective heat transfer and fluid/nanofluid flow through a sinusoidal wavy-channel inside a porous medium is performed through the optimization of entropy generation. The fluid flow in porous media is considered to be laminar and Darcy–Brinkman–Forchheimer model has been utilized. The obtained results were compared with the corresponding numerical data in order to ensure the accuracy and reliability of the numerical procedure. As a result, increasing the Darcy number leads to the increased portion of thermal entropy generation as well as the decreased portion of frictional entropy generation in all configurations. Moreover, configuration with wavelength of 10 mm, amplitude of 0.5 mm and phase shift of 60° was selected as an optimum geometry for further investigations on the addition of nanoparticles. Additionally, increasing trend of average Nusselt number and friction factor, besides the decreasing trend of performance evaluation criteria (PEC) index, were inferred by increasing the volume fraction of the nanofluid (Al_2_O_3_ and CuO).

## 1. Introduction

Achieving improved heat transfer in tubes and channels are of great significance, especially in industrial and technological applications from the energy saving viewpoint. Hence, numerous studies have been performed on evaluating the flow field and heat transfer in diverse configurations [1]. Among the proposed procedures for ameliorating the thermal performance in various systems, the utilization of wavy-wall channels is considered to be an efficient approach with the application in heat exchangers, heat sinks, and solar collectors. This issue has been addressed in several investigations [2,3,4,5,6,7]. Additionally, the heat transfer in wavy-wall channels have been studied extensively considering their various applications [8,9,10,11,12,13,14,15,16]. In an experimental study conducted by Rush et al. [17], the flow field and heat transfer characteristics were evaluated in corrugated wavy-wall minichannels. They introduced geometrical features of the channel and the Reynolds numbers as the most influential parameters on the local Nusselt number and, consequently, the local heat transfer. In a numerical investigation, Wang and Chen [18] investigated the heat transfer and forced convection of nanofluid flow (in laminar flow regime) within sinusoidal wavy-wall minichannels. They stated the effectiveness of the wavelength, amplitude ratio, and Reynolds number in improving the heat transfer in these channels. Zhang and Che examined the influence of corrugation profile on flow field and heat transfer in the cross-corrugated walls [19]. Their results declared the higher Nusselt number and friction factor in trapezoidal channels compared to the elliptical ones. A numerical study on the one-sided corrugated plate (in turbulent flow regime) was performed by Naphon and Kornkumjayrit [20]. According to their results, the superiority of the studied geometry in improving the heat transfer was concluded. In another numerical investigation, Khoshvaght-Aliabadi [21] studied the fluid flow and heat transfer in sinusoidal-corrugated channels with the addition of nanofluid. Regarding their results, Nusselt number and friction factor high were affected by channel height and wave amplitude, respectively.

The use of porous media in industrial equipment has been considered to be a practical procedure for ameliorating the heat transfer, which has attracted the attentions of many researchers [22,23,24,25,26,27,28]. The partial employment of the porous media in the core of a sinusoidal channel was explored by Akbarzadeh and Maghrebi [29]. They assessed the hydrothermal performance of different geometries and metal foam characteristics on local thermal non-equilibrium method. The heat transfer in a counter flow sinusoidal parallel-plate heat exchanger was numerically examined by Arasteh et al. [30]. The divergent sections of the desired heat exchanger were filled partially with the metal foam porous media. They reported an enhanced heat transfer rate, efficiency and overall heat transfer coefficient up to 19.2%. In recent years, the simultaneous application of porous media and nanofluids has proved to be effective in improving the heat transfer performance. In numerical and analytical investigations, Hajipour and Dehkordi [31] studied the fully-developed mixed-convective heat transfer of nanofluid flow inside the parallel-plate channel. The studied channel was filled partially with porous medium. In addition, the momentum and thermal energy equations were characterized by the corresponding Darcy–Brinkman–Forchheimer and different viscous dissipation models. The simultaneous application of nanofluid and porous medium in throttle area of the sinusoidal channel was explored by Nazari and Toghraie [32]. They utilized local thermal equilibrium model for the evaluation of fluid flow and heat transfer in the desired system. Their outcomes declared the higher improvement of heat transfer parameters in convex areas, rather than the concave areas of the throttle.

The addition of nanofluid to the fluid flow of channels has been the subject of several investigations [33,34,35,36,37,38,39]. In a numerical survey by Heidary and Kermani [40], the heat transfer and nanofluids flow within a corrugated wavy-wall channel were investigated. They stated the enhanced Nusselt number by increasing the Reynolds number, nanoparticle volume fraction, and wave amplitude. The influence of groove angel in a corrugated duct with $SiO_{2}$/water nanofluid on heat transfer and flow properties was assessed by Sadripour [41]. Their results revealed the high dependency of hydraulic and thermal efficiencies of the desired exchanger to the variation of grooves angles. In addition, the best performance evaluation criteria (PEC) index corresponded to the configuration with the duct angle of 30°, volume fraction of 4%, and nanoparticle size of 40 nm. Ho et al. [42] carried out a study on the forced convection heat transfer of Al_2_O_3_-water nanofluid. They stated the higher effectiveness of the nanofluid with volumetric concentration of 1%, rather than 2%, according to the higher variation of dynamic viscosity by temperature changes. They also reported 70% enhancement in convective heat transfer coefficient by the utilization of Al_2_O_3_-water nanofluid with the concentration of 1%. The numerical study of CuO nanofluid in a micro-tube was performed by Akbari et al. [43] by the use of a two-phase model. As their results depicted, the addition of nanoparticles would result in a reduction of Nusselt number and friction factor. The effect of several variables, including mass flow rate of the nanofluid, concentration of the nanoparticle, as well as geometrical parameters in wavy minichannels were examined by Aliabadi and Sahamiyan [44]. In their study, they evaluated the heat transfer and pumping power for the Al_2_O_3_-water flow under different conditions.

The other advantageous parameter that is helpful for achieving more comprehensive understanding of the quality of available energy and optimal thermodynamic conditions is known as entropy generation [45,46,47,48,49,50,51]. The minimization of entropy generation is proposed as a solution in several researches in order to optimize the thermodynamic systems [52,53,54,55,56,57]. Similar to the conducted researches on the field of evaluating the heat transfer in wavy-wall channels, the entropy generation can be investigated in both natural and forced convections. Sheremet et al. examined the entropy generation of natural convection heat transfer [58]. For this purpose, they utilized computational fluid dynamics (CFD) procedure. Their studied system consisted of Cu/water nanofluid in a cavity with corrugated perpendicular wall. In a numerical investigation by Cho et al. [59], the finite volume method was utilized for the simulation of entropy generation and natural convective heat transfer in a horizontal enclosure with wavy walls. They also investigated the effect of different metal oxide nanofluids. Different indexes, such as heat transfer, pressure drop, and entropy generation in the heat exchanger with sinusoidal wavy-wall and a porous insert with the nanofluid flow were examined by Akbarzadeh et al. [60] The calculation of entropy generation in a wavy heat exchanger with nanofluid flow was performed by Esfahani et al. [61]. They used two-dimensional (2D) simulation by ANSYS-FLUENT software for this aim. In a study by Dormohammadi et al. [62], entropy generation minimization technique was used for optimizing the mixed convective heat transfer in a wavy channel with nanofluid flow. Siavashi et al. performed the numerical investigation of the flow characteristics, heat transfer, and entropy generation in the annular pipe that were filled fully or partially with the porous media and nanofluid flow [63]. They utilized two-phase mixture model for the investigations and declared the dependency of the performance and entropy generation to the configuration parameters, nanoparticles concentration, and Reynolds number. Moreover, they disclosed the existence of an optimum thickness of the porous media for each nanofluid flow in a porous medium at a specific Reynolds number according to the thermodynamics second law.

Based on the available literature, the impact of simultaneous application of wavy-wall and porous medium, as well as their individual usage on heat transfer and fluid flow have been investigated in several studies. However, to the best of author’s knowledge, only a few studies concerning the laminar forced convective heat transfer of nanofluids flow as well as the calculation of entropy generation in a wavy-wall minichannel with porous media has been conducted. Therefore, in the current study, it is aimed to conduct a numerical investigation to evaluate the heat transfer, nanofluid flow, and entropy generation in a sinusoidal-wavy minichannel with porous medium. Moreover, the dependency of the average Nusselt number, friction factor, PEC index, and thermal/frictional entropy generation on geometrical characteristics and porosity would be evaluated. Furthermore, the impact of adding nanofluids (Al_2_O_3_ and CuO) to the pure fluid and volume fraction is also performed.

## 2. Materials and Methods

### 2.1. Geometrical Specifications of Physical Model

**Figure 1:**
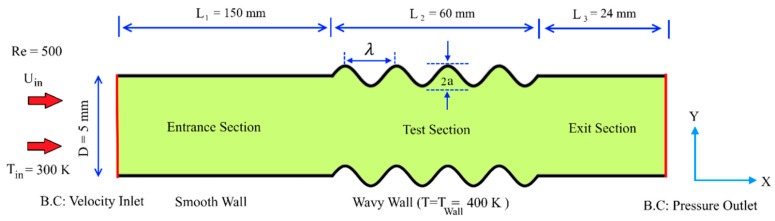
Schematic geometry of the problem.

The schematic representation of the studied arrangement is depicted in Figure 1, which shows the system consisting of three main sections; entrance, test and exit sections. The entrance section with the length of 150 mm was utilized in order to ensure the fully developed fluid flow in the test section. This section was followed by the test section with the length of 60 mm, which consisted of a two-dimensional minichannel with the opening height of D=5 mm and two sinusoidal-wavy walls. Finally, the exit section with the length of 24 mm was implemented in order to avoid the development of adverse pressure according to the subsequent influence of the heat transfer and fluid flow properties in computational domain. In this study, the effect of wave amplitude, wave length, and phase shift were evaluated. For investigating the influence of wave amplitude, three constant values of a=1.5, 1, and 0.5 mm, as well as two variable values (with decreasing and increasing trend) were considered. In addition, the similar procedure was considered for the wavelength; three constant values of λ=10, 12, and 15 mm and two variable values (with decreasing and increasing trend). Moreover, the effect of phase shift was evaluated in five values of φ=0°, 30°, 60°, 90°, and 180° among lower and upper wavy-walls. It should be noted that the outlet boundary conditions are pressure outlet.

### 2.2. Governing Equations

The mathematical formulations include mass, momentum, and energy conservation equations in a 2D steady-state flow, as below [62]:

(1) $$\frac{\partial u}{\partial x}+\frac{\partial v}{\partial y}=0$$

(2) $$\frac{1}{\varepsilon^{2}}\left(u\frac{\partial u}{\partial x}+v\frac{\partial u}{\partial y}\right)=-\frac{1}{\rho_{f}}\frac{\partial p}{\partial x}+\frac{\vartheta_{m}}{\varepsilon }\left(\frac{\partial^{2}u}{\partial x^{2}}+\frac{\partial^{2}u}{\partial y^{2}}\right)-\frac{\vartheta_{f}\;u}{K}-\frac{\vartheta_{m}C_{d}\left|u\right|}{\sqrt{K}}u$$

(3) $$\frac{1}{\varepsilon^{2}}\left(u\frac{\partial v}{\partial x}+v\frac{\partial v}{\partial y}\right)=-\frac{1}{\rho_{f}}\frac{\partial p}{\partial y}+\frac{\vartheta_{\mathrm{fm}}}{\varepsilon }\left(\frac{\partial^{2}v}{\partial x^{2}}+\frac{\partial^{2}v}{\partial y^{2}}\right)-\frac{\vartheta_{m}\;v}{K}-\frac{\vartheta_{m}C_{d}\left|v\right|}{\sqrt{K}}v$$

(4) $$u\frac{\partial T}{\partial x}+v\frac{\partial T}{\partial y}=\alpha_{m}\left(\frac{\partial^{2}T}{\partial x^{2}}+\frac{\partial^{2}T}{\partial y^{2}}\right)$$

For dimensionless form of equations, some variables are presented, as below.

(5) $$\;X=\frac{x}{D_{h}},\;Y=\frac{y}{D_{h}},\;U=\frac{u}{U_{\mathrm{in}}},\;V=\frac{v}{U_{\mathrm{in}}},\;\theta =\frac{T-T_{\mathrm{in}}}{T_{w}-T_{\mathrm{in}}}\;\alpha_{m}=\frac{k_{m}}{{\left(\rho C_{p}\right)}_{m}},\;P=\frac{p}{\rho_{f}U_{in}^{2}},\;C_{d}=\frac{1.75}{\sqrt{150}\varepsilon^{\frac{3}{2}}},\;T_{b}=\frac{\int_{0}^{D_{h}}uTdy}{\int_{0}^{D_{h}}udy}$$

(6) Re=ρfUinDhμf, Nu=Dh(∂T∂y)wallTwall−Tb, Da=KDh2, Pr=ϑfαf

In Equation (6), Re, Nu, Da, and Pr represent, Reynolds, Nusselt, Darcy, and the Prandtl numbers, respectively. In order to calculate the effective thermal conductivity of the applied fluid (based on the volumetric linear models) in porous media, the following equations were utilized [64]:

(7) $$k_{m}=\left(1-\varphi \right)k_{f}+{\varphi k}_{\mathrm{np}}$$

(8) $$k_{eff}=\left(1-\varepsilon \right)k_{p}+\varepsilon k_{m}$$

Additionally, the hydraulic diameter of corrugated wavy-wall has been presented as:

(9) $$D_{h}=2D+2a$$

By substituting non-dimensional parameters into Equations (1)–(4), non-dimensional form of equations will be achieved.

(10) $$\frac{\partial U}{\partial X}+\frac{\partial V}{\partial Y}=0$$

(11) 1ε2(U∂U∂X+V∂U∂Y)=−∂P∂X+1Re.ε(∂2U∂X2+∂2U∂Y2)−URe Da−εCd|U|DaU

(12) 1ε2(U∂V∂X+V∂V∂Y)=−∂P∂Y+1Re.ε(∂2V∂X2+∂2V∂Y2)−VRe Da−εCd|V|DaV

(13) (U∂θ∂X+V∂θ∂Y)=−1Re.Pr(ρcp)f(ρcp)m(∂2θ∂X2+∂2θ∂Y2)

### 2.3. Thermo-Physical Properties of Nanofluids

Extensive investigations have been conducted for the determination of the physical characteristics of nanofluids. In this study, the desired nanofluid was prepared by the dispersion of copper and aluminum oxide particles in water (base fluid). The physical characteristics of the desired nanofluid can be calculated through the following equations:

(14) $$\rho_{m}=\left(1-\phi \right)\rho_{f}+\phi \rho_{\mathrm{np}}$$

(15) $${\left(\rho \beta \right)}_{m}=\left(1-\phi \right){\left(\rho \beta \right)}_{f}+\phi {\left(\rho \beta \right)}_{\mathrm{np}}$$

(16) $${\left({\rho c}_{p}\right)}_{m}=\left(1-\phi \right){\left({\rho c}_{p}\right)}_{f}+\phi {\left({\rho c}_{p}\right)}_{\mathrm{np}}$$

In Equations (14)–(16), the indices of m, f, and np are corresponding to the respective characteristics of mixture, pure fluid, and nanoparticles. There are several models regarding dynamic viscosity that can be used, depending on the problem solving conditions. In this work, after detailed reviewing the proposed models, it is found that some researchers have been used the following experimental model for the volume fraction in the selected range (based on [65]). The nanofluid viscosity can be defined through the following equations [65]:

(17) $$\mu_{\mathrm{np}}=\mu_{f}\left(-0.188+537.42\phi \right)$$

(18) $$\mu_{m}=\left(1-\phi \right)\mu_{f}+\phi \mu_{\mathrm{np}}$$

### 2.4. Entropy Generation Analysis

The entropy generation can be attributed to the irreversibility as a consequence of heat transfer (thermal part) and fluid flow friction (frictional part). Several investigations have been dedicated to minimizing the entropy generation through the second law of thermodynamics. The entropy generation rate can be determined regarding the procedure introduced in Reference [66], as follows:

(19) $$S_{\mathrm{gen}}^{\prime \prime \prime }=S_{\mathrm{gen},f}^{\prime \prime \prime }+S_{\mathrm{gen},T}^{\prime \prime \prime }$$

The following relation is used for the calculation of $S_{\mathrm{gen},T}^{\prime \prime \prime }$ in porous media:

(20) $$S_{\mathrm{gen},T}^{\prime \prime \prime }=\frac{k_{m}}{T_{\mathrm{in}}^{2}}\left[{\left(\frac{\partial T}{\partial x}\right)}^{2}+{\left(\frac{\partial T}{\partial y}\right)}^{2}\right]$$

Additionally, the friction-induced generation of entropy in porous media is determined by:

(21) $$S_{\mathrm{gen},f}^{\prime \prime \prime }=\frac{\mu_{m}}{T_{\mathrm{in}}}\left[2{\left(\frac{\partial u}{\partial x}\right)}^{2}+2{\left(\frac{\partial v}{\partial y}\right)}^{2}+{\left(\frac{\partial u}{\partial y}+\frac{\partial v}{\partial x}\right)}^{2}\right]+\frac{\mu_{m}}{{\mathrm{KT}}_{\mathrm{in}}}\left[u^{2}+v^{2}\right]$$

(22) $$S_{\mathrm{gen}}=\int S_{\mathrm{gen}}^{\prime \prime \prime }\mathrm{dV}$$

In the above equations, $S_{\mathrm{gen},T}$ and $S_{\mathrm{gen},f}$ represent the entropy generation induced by heat transfer and fluid friction, respectively.

### 2.5. Boundary Conditions

The steady laminar flow was considered for the entrance flow to the minichannel, as depicted in Figure 1. The Reynolds number of this flow can be calculated through Equation (6) (Re=500). In addition, constant temperatures of $T_{w}=400\;K$ were considered for both wavy walls, while the inlet flow temperature was assumed to be $T_{in}=300\;K$. In addition, steady-state parameters were considered for the heat transfer and fluid flow. Furthermore, fully saturated condition (ε=0.9) with Newtonian and incompressible fluid were presumed for the porous medium. The thermo-physical properties of operating fluid and porous medium were maintained constant. The local thermal equilibrium between the operating fluid and porous medium were also established. The thermo-physical properties of the used water/nanoparticles and porous medium are provided in Table 1 and Table 2, respectively.

### 2.6. Numerical Simulation

For the 2D numerical simulations, the commercial software of ANSYS FLUENT was utilized. The equations of energy and momentum were discretized through the second-order upwind method. The line-by-line procedure was implemented for solving the obtained equations iteratively. Moreover, velocity and pressure fields were related to each other by the use of SIMPLE algorithm (semi-implicit method for pressure-linked equations). The considered divergence criteria for the conservation equations of mass, energy and momentum was equal to $10^{-5}$. The under-relaxation factors for energy, momentum and pressure equations were considered to be the respective values of 1, 0.7, and 0.3 for maintaining the stability of the solution.

### 2.7. Governing Equations

**Figure 2:**
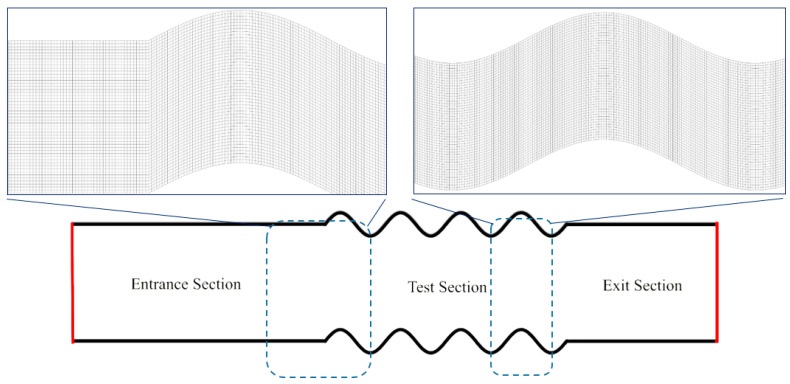
The generated grid for the current work.

The generating grids is regarded as the preliminary and important steps in numerical simulation while considering the subsequent effect on computational time, convergence and solution results. The generated grid for the current problem is depicted in Figure 2. Better performance would be achieved through the use of regular gridding as compared to the irregular counterpart. Finer and regular gridding with radially incrementing elements should be implemented in the elements adjacent to the wall for evaluation of steep gradient of physical features.

**Figure 3:**
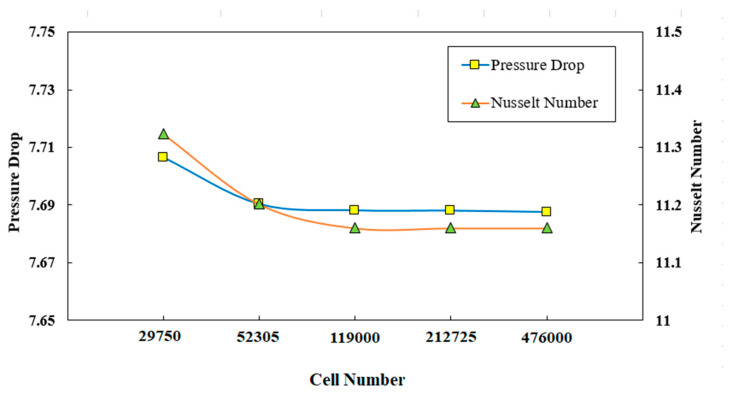
Variations of pressure drop and Nusselt number with diverse cell numbers.

The grid independency of the results was also evaluated and provided in Figure 3. As can be seen in this figure, grid 4 revealed approximately 0.5% deviation for the Nusselt number and pressure drop. The results for diverse grid sizes are also provided in Figure 3. According to this figure, the grid with 119,000 nodes would be efficient when considering both accuracy and computational time.

### 2.8. Model Validation

**Figure 4:**
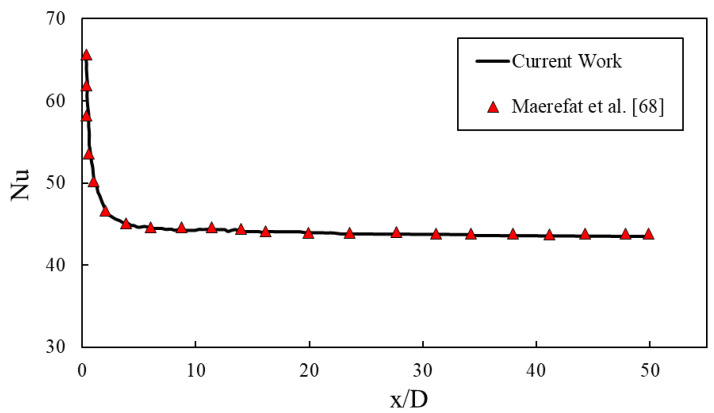
Comparison between the present numerical data and the numerical data of Maerefat et al. [68].

The present results were compared with the numerical data of Maerefat et al. [68] for the same operating conditions to validate the simulation procedure. The comparison results for Nusselt number in sinusoidal minichannel revealed an acceptable agreement, as provided in Figure 4. The same operating conditions reported by Maerefat et al. [68] are adopted here for the model validation. It is necessary to mention that, parameters of D, x, and Nu in Figure 4 are diameter, length of the pipe, and local Nusselt number along the length of the pipe.

## 3. Results and Discussion

### 3.1. Effect of Geometrical Properties on Average Nusselt Number

**Figure 5:**
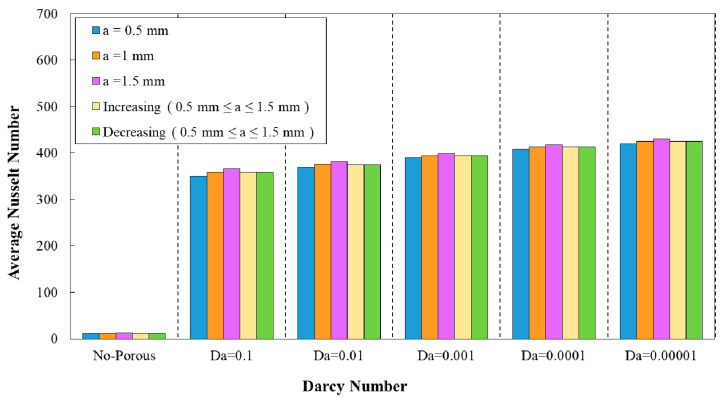
Comparison of average Nusselt number for diverse Darcy numbers in various wave amplitudes (λ=12 mm, φ=0°).

**Figure 6:**
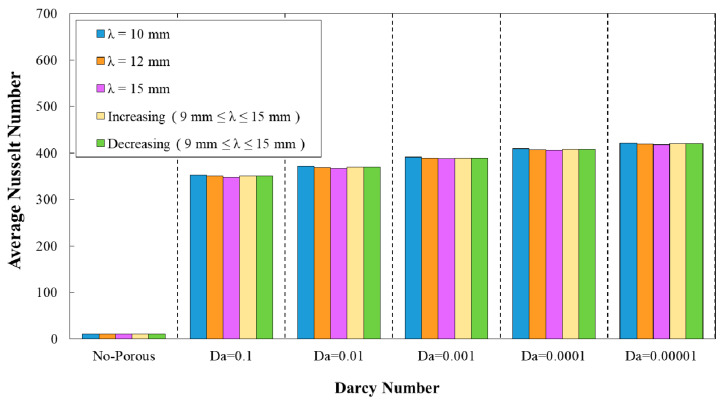
Comparison of average Nusselt number for diverse Darcy numbers with various wavelengths (a=0.5 mm, φ=0°).

**Figure 7:**
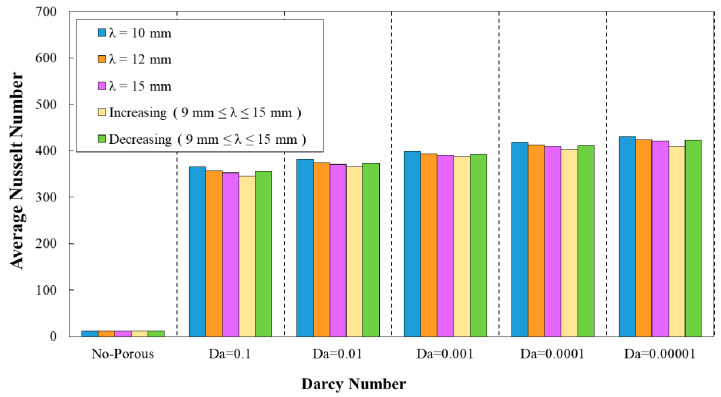
Comparison of average Nusselt number for diverse Darcy numbers with various wavelengths and decreasing wave amplitude (φ=0°).

**Figure 8:**
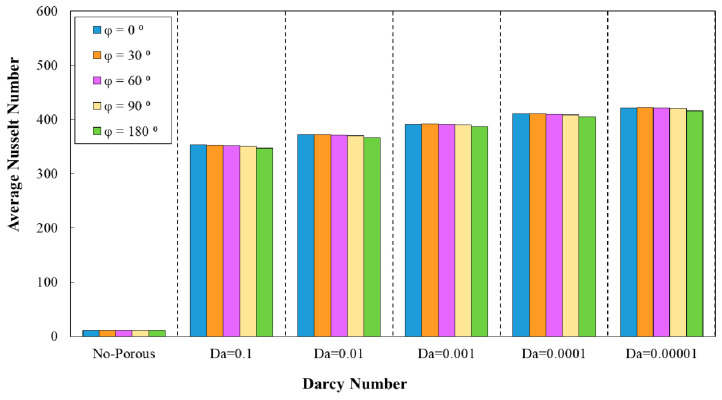
Comparison of average Nusselt number for diverse Darcy numbers with various phase shifts and wavelength of (a=0.5 mm, λ=10 mm).

**Figure 9:**
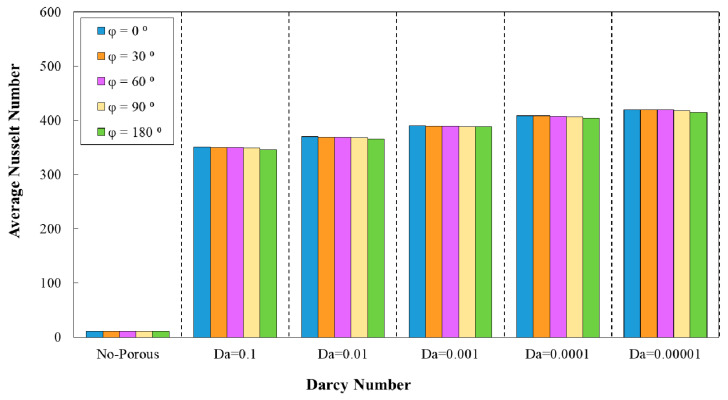
Comparison of average Nusselt number for diverse Darcy numbers with various phase shifts and increasing wavelength.

The diagram of average Nusselt number against different Darcy numbers are plotted in Figure 5, Figure 6, Figure 7, Figure 8 and Figure 9 in terms of wave amplitude, wavelengths, and phase shifts. Based on Figure 5, Figure 6, Figure 7, Figure 8 and Figure 9, the enhanced average Nusselt number would be achieved by the decrement of Darcy number from 0.1 to 0.0001. In Figure 5, for the desired diagram was plotted for sinusoidal minichannel with the wavelength of λ=12 mm and phase shift of φ=0°. The ameliorated average Nusselt number approximately up to 400 as a result of increasing flow velocity is evident from this figure. As it is clear, the higher average Nusselt number was achieved for all of the studied configurations of corrugated wavy minichannel regarding the introduction of superior disturbance and formation of thinner boundary and the subsequent higher temperature gradients. The highest heat transfer was in accordance with the configuration with wave amplitude of a=1.5 mm.

The diagram of the average Nusselt number against Darcy number at wave-amplitude of a=0.5 mm for diverse wavelengths is plotted in Figure 6. As it is obvious from this figure, the reduction of Darcy number was followed by an increment in the average Nusselt number. The smaller Darcy number represents the lower medium permeability, which resulted in an improved heat transfer and increased average Nusselt number. In addition, the highest average Nusselt number was belonged to the wavy minichannel with the wavelength of λ=10 mm. Moreover, Figure 7 represents the average Nusselt number against Darcy number for the decreasing trend of wave amplitude. Regarding this figure, the wavy tube with the wavelength of λ=10 mm attained the highest average Nusselt number value when compared to other configurations.

Figure 8 represent the variation of average Nusselt number against Darcy number in five different phase shifts of wavy minichannel. The amplitude and the wavelength were considered to be the constant values of a=0.5 mm and =12 mm, respectively. In addition, the variation of average Nusselt number against Darcy numbers for five phase shifts of wavy minichannel with the amplitude of a=0.5 mm and augmenting wavelength is depicted in Figure 9. Regarding these two figures, the maximum average Nusselt number was achieved in the phase shift of φ=0°, which was followed by the phase shifts of φ=30°, 60°, 90°, and 180°, respectively.

### 3.2. Effect of Geometrical Properties on Friction Factor

The friction factor for fully developed flow can be calculated from Equation (23):

(23) $$f=\frac{2}{\left(\frac{L}{D_{h}}\right)}\frac{\Delta p}{{\rho U}_{\mathrm{in}}^{2}}$$

**Figure 10:**
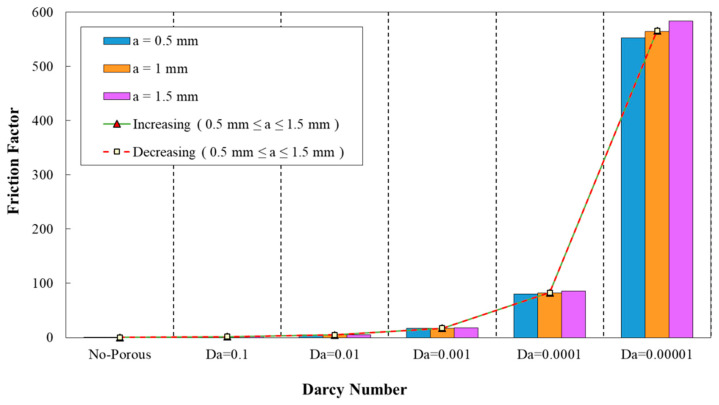
Comparison of friction factor for diverse Darcy numbers in various wave-amplitudes, (λ=12 mm, φ=0°).

**Figure 11:**
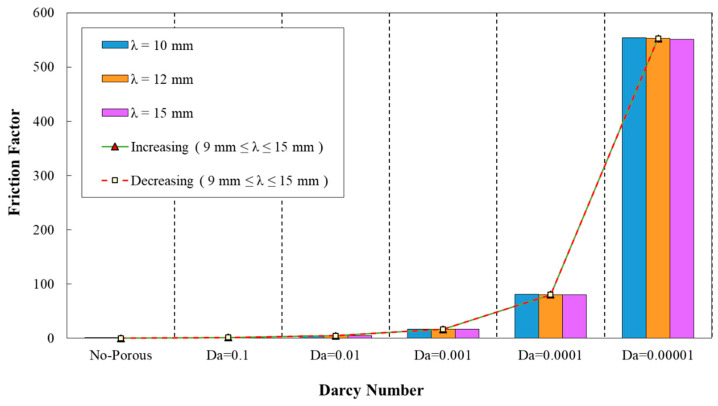
Comparison of friction factor for diverse Darcy numbers with various wavelengths (a=0.5 mm, φ=0°).

**Figure 12:**
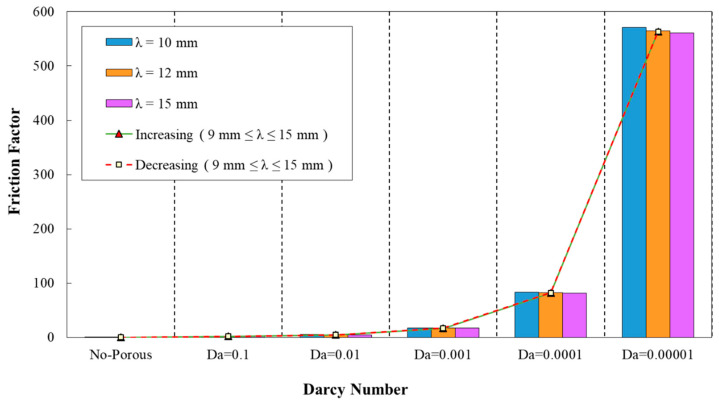
Comparison of friction factor for diverse Darcy numbers with various wavelengths and decreasing wave amplitude (0.5 mm≤a≤1.5 mm).

**Figure 13:**
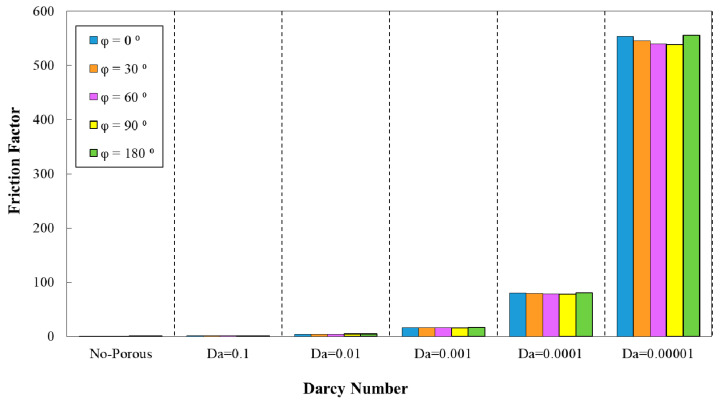
Comparison of friction factor for diverse Darcy numbers with various phase shifts (a=0.5, λ=10 mm).

**Figure 14:**
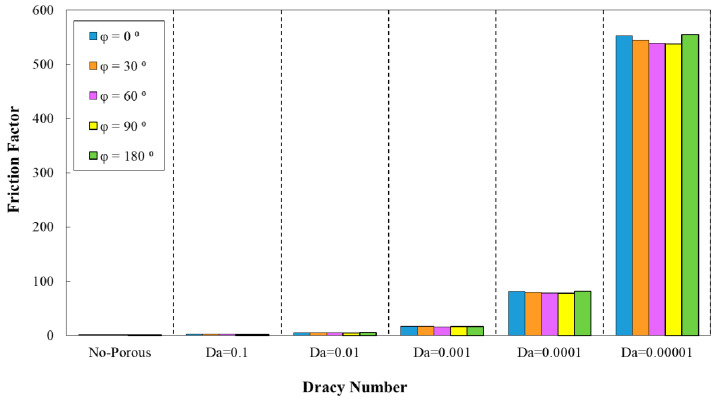
Comparison of friction factor for different Darcy numbers with various phase shifts and increasing wavelength.

In the above relation, Δp, L, and $D_{h}$ are the respective values for pressure drop, minichannel length, and hydraulic diameter. The effects of Darcy number on friction factor for different parameters are represented in Figure 10, Figure 11, Figure 12, Figure 13 and Figure 14. The augmenting trend of friction factor with the reduction of Darcy numbers is obvious from Figure 10, Figure 11, Figure 12, Figure 13 and Figure 14, while it showed a reducing behavior by increasing the porosity from Da= 0.0001 to Da= 0.1 at a constant wave amplitude. Various constant and variable wavy amplitudes in the diagram of friction factor versus Darcy number are analyzed in Figure 10. Regarding this figure, the highest friction factor was attributed to the wavy amplitude of α=1.5 mm.

The friction factor coefficient variation against Darcy number for different wavelength are plotted in Figure 11. As it is obvious from this figure, the wavelength of λ=10 mm was corresponding to the maximum value of friction factor, while the wavelength of λ=15 mm attained the minimum friction factor coefficient. In addition, the variation of friction factor against Darcy numbers for various wavelengths and decreasing wave amplitude is depicted in Figure 12. This figure reveals the minimum friction factor for the configuration with the wavelength of λ=15 mm. From Figure 11 and Figure 12, it can be inferred that, in all of the studied configurations, the friction factor showed the reducing behavior by increasing Darcy number from 0.0001 to 0.1.

Figure 13 depicts the variation of friction factor in the desired minichannel for different phase shifts and wavelength of λ=10 mm. The maximum friction factor value was associated with the phase shift of φ=180° as it is obvious from this figure. However, the configuration with the phase shift of φ=90° had attained the minimum friction factor value. Moreover, in Figure 14, the variations of the friction factor in corrugated minichannel for various phase shifts and increasing wavelength are represented. The highest and lowest friction factor values were related to the phase shift of φ=180° and 90°, respectively.

### 3.3. Effect of Geometrical Properties on the PEC Index

The PEC index has been introduced for the calculation of fluid dynamic and thermal performances. This parameter is beneficial for evaluating the heat transfer performance and pumping power in corrugated wavy-wall minichannel with variable amplitudes. This factor can be determined by the use of estimated friction factor and average Nusselt numbers coefficient, as follows [13]:

(24) $$\mathrm{PEC}=\frac{\left(\frac{{\mathrm{Nu}}_{\mathrm{av}.w}}{{\mathrm{Nu}}_{\mathrm{av},s}}\right)}{{\left(\frac{f_{w}}{f_{s}}\right)}^{1/3}}$$

**Figure 15:**
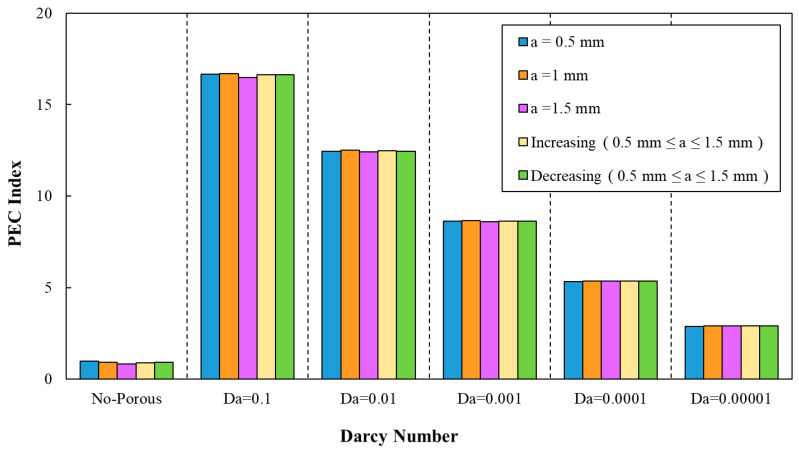
The variation of performance evaluation criteria (PEC) index against different Darcy numbers for various wave-amplitudes, (λ=12 mm, φ=0°).

**Figure 16:**
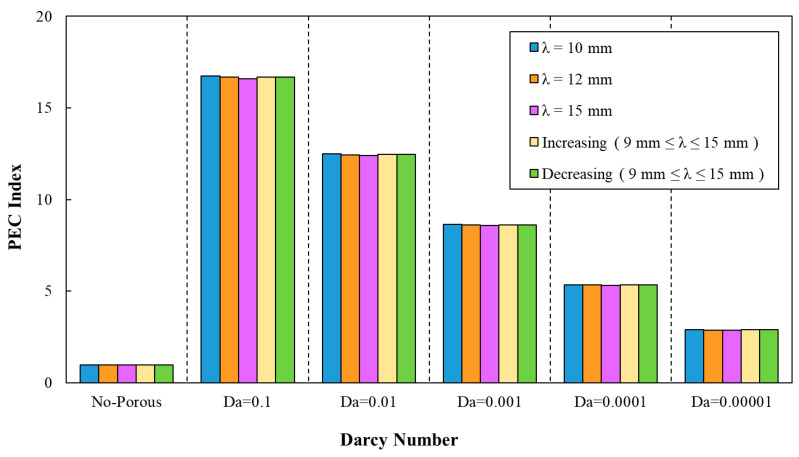
The variation of PEC index against different Darcy numbers for various wavelengths, (a=0.5 mm, φ=0°).

**Figure 17:**
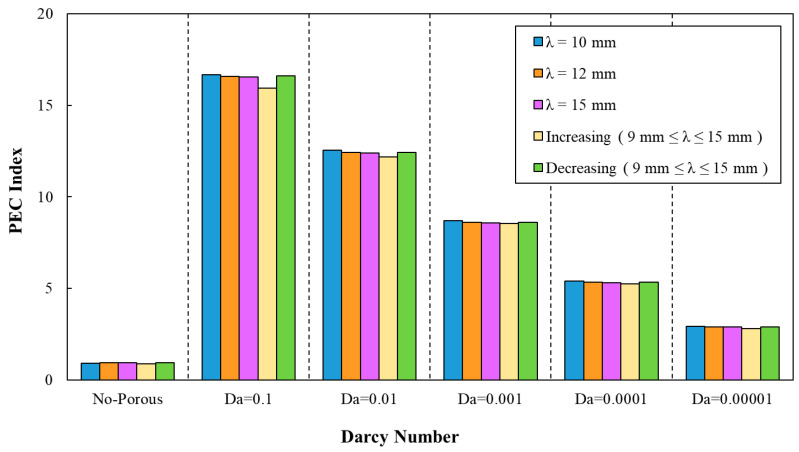
The variation of PEC index against different Darcy numbers for various wavelengths and decreasing wave amplitude.

**Figure 18:**
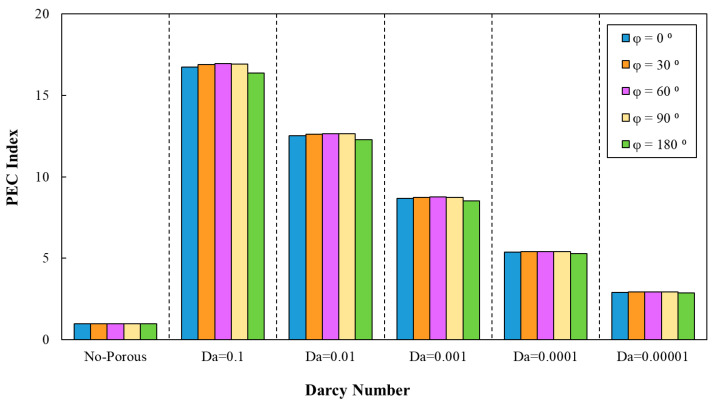
The variation of PEC index against different Darcy numbers for various phase shifts, (a=0.5 mm, λ=10 mm).

**Figure 19:**
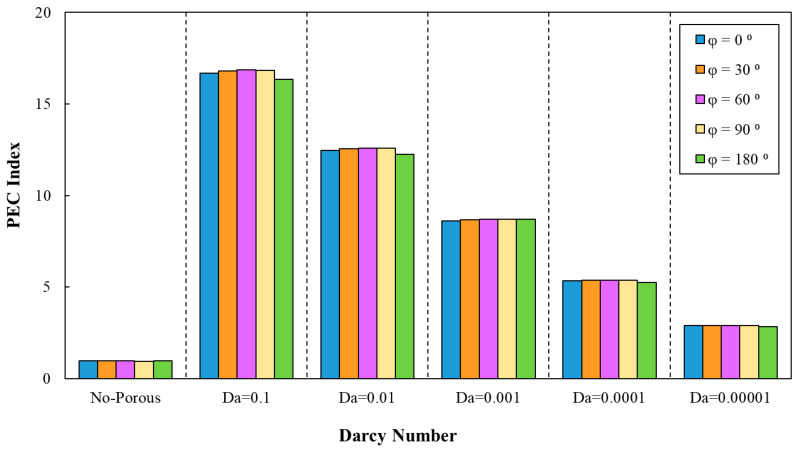
The variation of PEC index against different Darcy numbers for various phase shifts and increasing wavelength, (a=0.5 mm, 9 mm≤λ≤15 mm).

In which, ${\mathrm{Nu}}_{\mathrm{av}.w}$ and ${\mathrm{Nu}}_{\mathrm{av},s}$ as well as $f_{w}$ and $f_{s}$ represent the estimated average Nusselt number and friction factor for wavy and smooth minichannel, respectively. The variations of PEC index against Darcy number for different geometrical properties are provided in Figure 15, Figure 16, Figure 17, Figure 18 and Figure 19. Regarding these figures for all of the studied configurations, PEC index was ameliorated by increasing the Darcy number as a consequence of heat transfer coefficient effect on the flow. The highest PEC index was attributed to Da=0.1, as can be seen. The variation of PEC index versus Darcy numbers for different wave-amplitudes is plotted in Figure 15. From this figure, it can be realized that wavy amplitude of a=1 mm gained the maximum PEC index among all other configurations. Moreover, the application of wave-amplitude of a=0.5 mm, resulting in a higher Nusselt number.

The PEC index was determined by average Nusselt numbers and friction factor coefficients, as can be seen in Figure 16. Regarding this figure, a declining trend of PEC index by the increasing of Darcy number can be inferred. In addition, the determination of the PEC index from friction factor coefficients and average Nusselt numbers can be also inferred from Figure 17. The best configuration from the PEC index perspective belonged to the sinusoidal-wavy minichannel in porous media with the wavelength of λ=10 mm.

Similar to the previous cases, according to Figure 18, the PEC was determined for various phase shifts, a=0.5 mm and =10 mm. This figure states the enhancement of the PEC index in minichannel by increasing Darcy number. The highest PEC index (approximately 16.95) in the sinusoidal corrugated minichannels was associated with the configuration with the phase shift of φ=60°, wave amplitude of a=0.5 mm, and wavelength of λ=10 mm at Da=0.1. Hence, this configuration was selected as the optimal configuration from the PEC index viewpoint and used in further investigation of different nanofluids. In addition, the variation of PEC index against Darcy numbers for various phase shifts and increasing wavelength in the range of 9 mm≤λ≤15 mm and a=0.5 mm is plotted in Figure 19. As can be inferred from Figure 19, the most efficient configuration of sinusoidal-wavy minichannel in porous medium was determined as the configuration with the constant wave amplitude of a=0.5 mm, augmenting wavelength and phase shift of φ=60°.

### 3.4. Effect of Geometrical Properties and Porosity on Entropy Generation

**Figure 20:**
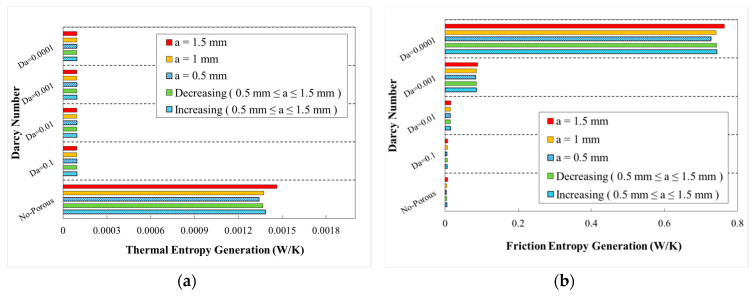
Entropy generation in terms of Darcy numbers at various wave-amplitudes for (**a**) thermal entropy generation and (**b**) friction entropy generation (λ=12 mm, φ=0°).

**Figure 21:**
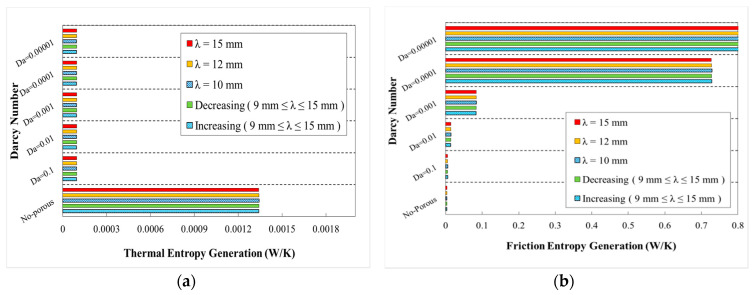
Entropy generation in terms of Darcy numbers at various wavelengths for (**a**) thermal entropy generation and (**b**) friction entropy generation (a=0.5 mm, φ=0°).

**Figure 22:**
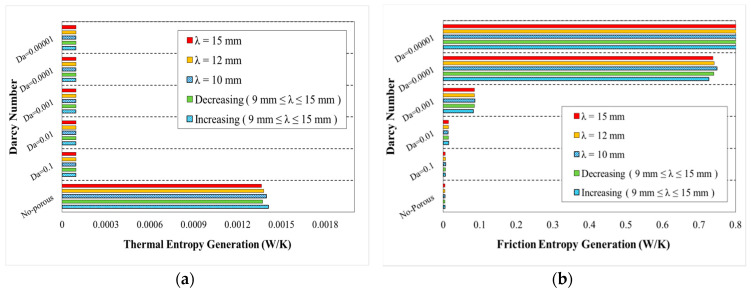
Entropy generation in terms of Darcy numbers at various wavelengths and decreasing wave-amplitude for (**a**) thermal entropy generation and (**b**) friction entropy generation.

**Figure 23:**
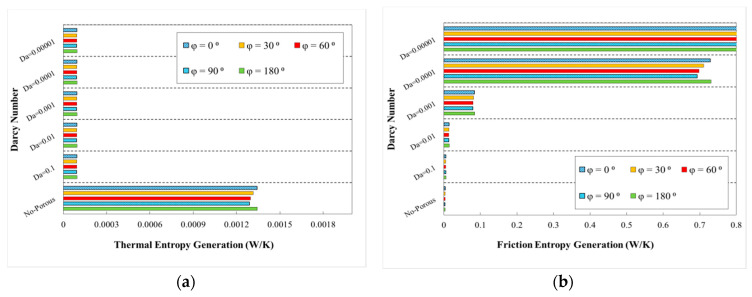
Entropy generation in terms of Darcy numbers at various phase shifts for (**a**) thermal entropy generation and (**b**) friction entropy generation, (a=0.5, λ=10 mm).

**Figure 24:**
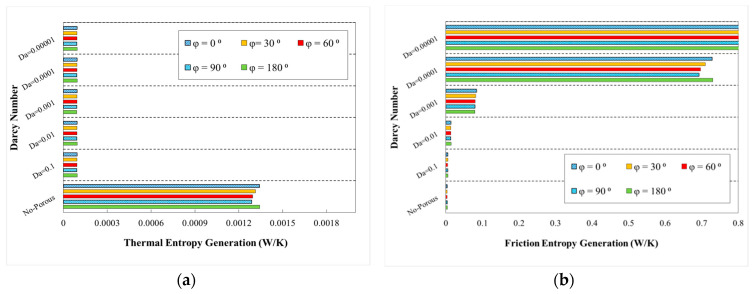
Entropy generation in terms of Darcy numbers at various phase shifts and increasing wavelength for (**a**) thermal entropy generation and (**b**) friction entropy generations.

The variations of generation of entropy according to the heat transfer (thermal entropy generation) and fluid flow (frictional entropy generation) are depicted in Figure 20, Figure 21, Figure 22, Figure 23 and Figure 24, respectively. These figures clarify the increasing and decreasing contributions of thermal and frictional entropy generations as a result of increased Darcy number, respectively. These figures also provide the profile of frictional entropy generation, which declares the higher contribution of frictional entropy generation at Da=0.00001 in comparison of the thermal entropy generation.

The variation of entropy generations for different wavelengths and wave amplitudes are depicted in Figure 21 and Figure 22, respectively. The geometrical properties of different configurations for the cases with constant wave amplitude of a=0.5 mm and reducing wave amplitude are depicted in Figure 21 and Figure 22, respectively. The approximately constant contribution of heat transfer in entropy generation by increasing wavelengths can be concluded regarding Figure 21. The variation of entropy generations against Darcy numbers for various phase shifts and decreasing wave amplitude is plotted in Figure 22. From this figure, it can be found that the use of porous medium and increasing Darcy number at all cases would result in the increased share of entropy generation (thermal portion) besides a decreased share of entropy generation (frictional portion) in the overall generated entropy.

The respective thermal and frictional entropy generations in various phase shifts, wave amplitude of a=0.5 mm and wavelengths of λ=12 mm, as well as the increasing wavelengths are presented in the respective diagrams of Figure 23 and Figure 24. According to the obtained results, thermal entropy generations share was increased, while the frictional share was decreased by increasing the Darcy number. As it is obvious from these figures, frictional entropy generation attained the higher contribution in total entropy generation at Da=0.0001 and Da=0.00001. The highest thermal and frictional entropy generations for the desired minichannel was associated with the phase shift of φ=0° and φ=180°, wave amplitude of a=0.5 mm, and wavelength of λ=10 mm. Moreover, regarding Figure 24, the sinusoidal-wavy minichannel in porous medium with constant wave-amplitude of a=0.5 mm, augmenting wavelength and phase shift of φ=0° and φ=180°, are the most efficient configuration for thermal and frictional entropy generations in comparison of other cases, respectively.

### 3.5. Effect of Utilizing Nanoparticles in Base Fluid and Volume Fraction

**Figure 25:**
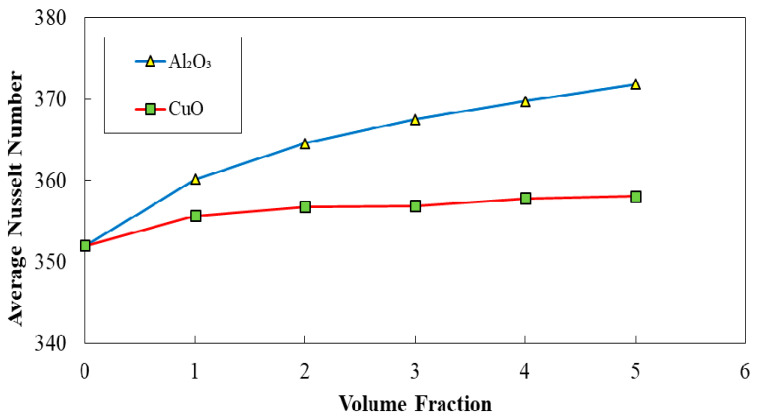
Comparison of average Nusselt number for diverse volume fraction in two case of ${\mathrm{Al}}_{2}O_{3}$ and CuO nanofluid (λ=10 mm,a=0.5 mm, and φ=60°).

**Figure 26:**
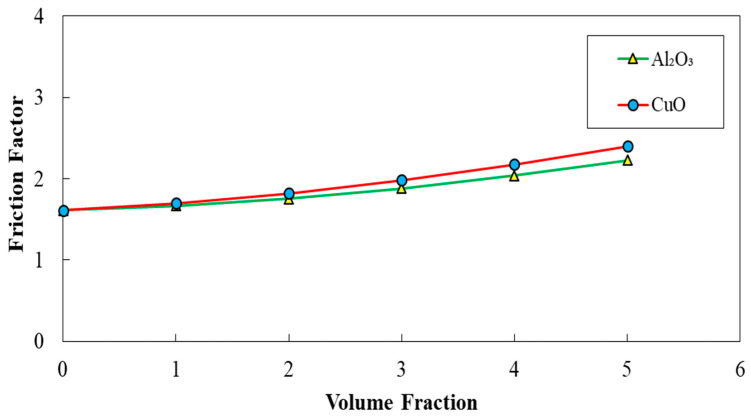
Comparison of friction factor for diverse volume fraction in two case of ${\mathrm{Al}}_{2}O_{3}$ and CuO nanofluid, (λ=10 mm,a=0.5 mm, and φ=60°).

**Figure 27:**
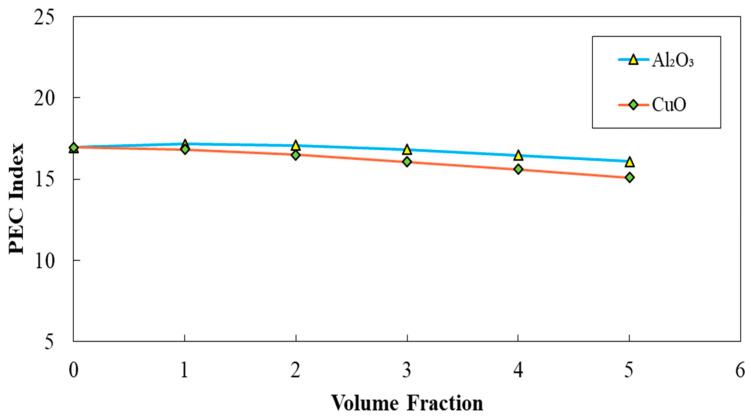
Comparison of PEC index for diverse volume fraction in two case of ${\mathrm{Al}}_{2}O_{3}$ and CuO nanofluid, (λ=10 mm,a=0.5 mm, and φ=60°).

The corrugated minichannels with a=0.5 mm, λ=10 mm, and φ=60° introduced as the most efficient configuration in terms of Nusselt number, friction factor, and PEC index among all studied ones, according to the obtained results in previous sections. Hence, this configuration was selected for conducting the further investigations on the addition of nanoparticles (Al_2_O_3_ and CuO) to the pure fluid. The diagrams of average Nusselt number, friction factor, and PEC index in terms of the volume fraction of two-phase nanofluid for two different nanofluids (Al_2_O_3_ and CuO nanofluid) are represented in Figure 25, Figure 26 and Figure 27, respectively. Regarding these figures, by augmenting the volume fraction of the nanoparticles, an increased average Nusselt number, and friction factor besides the decreased PEC index can be realized. The highest amelioration in Nusselt number was reported for the case of using Al_2_O_3_ nanofluid regarding the thermo-physical properties.

## 4. Conclusions

In this study, the influence of geometrical variables on the forced convective heat transfer and entropy generation of the minichannels with the simultaneous use of corrugation and porous media was investigated numerically. For this purpose, a 2D CFD scheme was proposed for modeling incompressible, steady, and laminar flow of nanofluid flow through the corrugated minichannel. The fluid flow in the porous media was characterized by Darcy–Brinkman–Forchheimer model. In addition, the effect of different parameters, including amplitude, wavelength, shift phase, and porosity of the wavy-wall were analyzed. The main findings of the current work can be summarized, as follows:

- Enhanced average Nusselt number was achieved by decreasing the Darcy number from 0.1 to 0.0001. The maximum average Nusselt number value was obtained as 430.91.
- Reducing behavior of friction factor with increasing the Darcy number from 0.0001 to 0.1 was observed at constant wavelength, amplitude and phase shift. Moreover, of the phase shifts of φ=180° and 90° corresponded to the maximum and the minimum friction factor, respectively.
- The PEC index of sinusoidal corrugated minichannel was maximized (maximum value of 16.95) for the phase shift of φ=60°, wave amplitude of a=0.5 mm, and wavelength of λ=10 mm at Da=0.1.
- The application of porous medium as well as increasing Darcy number at all cases, resulted in the increase and decrease in the respective thermal and frictional entropy generations.
- According to the obtained results, the geometrical properties of the most efficient configuration were λ=10 mm, a=0.5 mm, and φ=60°. This configuration was used for the investigations concerning the addition of nanoparticles (Al_2_O_3_ and CuO). Furthermore, the highest Nusselt number augmentation was stated for Al_2_O_3_ nanofluid, because of its thermo-physical characteristics.

## Figures and Tables

**Table 1:** Thermo-physical characteristics of water/nanoparticles (water/Al_2_O_3_-CuO) [32,63].

Properties	Water	Al_2_O_3_	CuO	Unit
$c_{p}$	4182	773	540	${J\;\mathrm{kg}}^{-1}{\;K}^{-1}$
*k*	0.6	40	18	${W\;m}^{-1}{\;K}^{-1}$
ρ	998.2	3880	6500	${\mathrm{kg}\;m}^{-3}$
μ	0.001003	-	-	${\mathrm{kg}\;m}^{-1}s^{-1}$
$D_{p}$	-	40	25	nm
β	$2.1\times 10^{-4}$	$0.85\times 10^{-5}$	$0.85\times 10^{-5}$	$K^{-1}$

**Table 2:** Thermo-physical characteristics of the porous medium [67].

Properties	Aluminum	Unit
$c_{p}$	871	$J\;kg^{-1}\;K^{-1}$
*k*	202	$W\;m^{-1}\;K^{-1}$
ρ	2719	$\mathrm{kg}\;m^{-3}$
*ε*	0.9	-

## Nomenclature

a	Wavy amplitude (m)
*C*	Specific Heat $({J\;\mathrm{kg}}^{-1}{\;K}^{-1}$)
*D*	Diameter (m)
*K*	Permeability of Porous Medium $\left(m^{2}\right)$
*k*	Thermal Conductivity $\left({W\;m}^{-1}{\;K}^{-1}\right)$
*L*	Length (m)
*P*	Dimensionless Pressure
*p*	Pressure (Pa)
*S*	Total Entropy
$S_{gen,T}$	Thermal Entropy Generation $\left({W\;K}^{-1}\right)$
$S_{gen,f}$	Friction Entropy Generation $({W\;K}^{-1}$)
*T*	Temperature (K)
U,V	Dimensionless Velocity
u,v	Velocity Components (${m\;s}^{-1}$)
X,Y	Dimensionless Coordinates
x,y	Dimensional Coordinates (m)
**Greek symbols**	
*α*	Thermal Diffusivity ($m^{2}{\;s}^{-1})$
*λ*	Wavelength (m)
*β*	Thermal Expansion Coefficient ($K^{-1}$)
*ε*	Porosity
*θ*	Dimensionless Temperature
*ϑ*	Kinematic Viscosity ($m^{2}{\;s}^{-1}$)
*μ*	Dynamic Viscosity (${\mathrm{kg}\;m}^{-1}s^{-1}$)
*ρ*	Density (${\mathrm{kg}\;m}^{-3}$)
φ	wavy-wall shift phase/degrees
ϕ	Volume Fraction
**Subscripts**	
av	Averaged
*f*	Pure Fluid
*s*	Smooth
*w*	Wavy
gen	Generation
*h*	Hydraulic
*m*	Mixture
np	Nanoparticle
**Abbreviations**	
CFD	Computational Fluid Dynamics
Da	Darcy Number
Nu	Nusselt Number
Pr	Prandtl Number
PEC	Performance Evaluation Criteria
Re	Reynolds Number
SIMPLE	Semi-Implicit Method for Pressure-Linked Equations
